# Regional versus general anaesthesia in elderly patients undergoing surgery for hip fracture: protocol for a systematic review

**DOI:** 10.1186/s13643-016-0246-0

**Published:** 2016-04-21

**Authors:** Joyce Yeung, Vanisha Patel, Rita Champaneria, Janine Dretzke

**Affiliations:** Institute of Inflammation and Ageing, College of Medical and Dental Sciences, University of Birmingham, Edgbaston, Birmingham, B15 2TT UK; Birmingham Clinical Trials Unit, Institute of Applied Health Research, College of Medical and Dental Sciences, University of Birmingham, Edgbaston, Birmingham, B15 2TT UK; Institute of Applied Health Research Public Health, Epidemiology and Biostatistics, Institute of Applied Health Research, College of Medical and Dental Sciences, University of Birmingham, Edgbaston, Birmingham, B15 2TT UK

## Abstract

**Background:**

With an ageing population, the incidence of hip fractures requiring surgery is increasing. Post-operative delirium is common following hip fracture surgery. Delirium is associated with high mortality and morbidity, poor long-term functional outcomes and institutionalisation. There is some evidence to suggest that perioperative intervention, specifically the anaesthetic technique employed, may reduce the incidence of delirium in this population. The aim of this systematic review is to investigate the impact of anaesthesia type on post-operative delirium.

**Method:**

We will conduct a systematic literature review using Embase, MEDLINE, CINAHL and the Cochrane Library (CENTRAL) bibliographic databases and the ZETOC and Web of Science websites. Authors of these trials will be invited to contribute unpublished data. PROSPERO register and clinical trial registers will also be searched to identify any ongoing reviews and trials. Eligible studies will assess the incidence of post-operative delirium in patients having regional or general anaesthesia for hip fracture surgery. The primary outcome of interest will be post-operative delirium; secondary outcomes will include mortality, measures of functional outcome, quality of life, length of hospital stay, discharge location and adverse events. Two reviewers will independently screen references identified by electronic literature searches. Two independent reviewers will extract data from studies fulfilling our inclusion criteria using a piloted data extraction form. Methodological quality and bias of included randomised controlled trials will be assessed using the ‘Cochrane Collaborations tool for assessing risk of bias’; for non-randomised studies, this will be assessed using the Newcastle-Ottawa scale. Data on similar outcomes will be pooled when possible. Where possible, meta-analysis will be undertaken using Review Manager (RevMan version 5.3) software.

**Discussion:**

This systematic review will provide an updated evidence base with which to guide clinical practice and research for this group of challenging patients. If the anaesthetic technique employed is shown to reduce the incidence of post-operative cognition dysfunction, then this may lead to a change in evidence-based practice, influence future guidelines and support further randomised controlled trial research. There is no known effective treatment for delirium, creating the urgent need for research into delirium prevention.

**Systematic review registration:**

PROSPERO CRD42015020166

**Electronic supplementary material:**

The online version of this article (doi:10.1186/s13643-016-0246-0) contains supplementary material, which is available to authorized users.

## Background

Delirium is a common neuropsychiatric syndrome defined in the Diagnostic and Statistical Manual of Mental Disorders (DSM V) as the disturbance of attention, awareness and cognition which develops over a short period of time and represents a change from baseline and tends to fluctuate during the course of the day [1, 2]. Patients with hip fractures have a high incidence of post-operative delirium of 32–53.3 % due to physiological and psychological stress from injury, pain, analgesia and surgery [3–5].

Post-operative delirium delays time to mobilise and discharge and increases the need for social input. Beyond the initial recovery period, delirium is associated with poor long-term functional outcomes, institutionalisation, anxiety and depression [6–14]. Some studies have also reported higher mortality whilst others reported that higher mortality was only seen in patients with pre-existing dementia. It remains unclear whether poor outcomes are associated with underlying dementia and neuroinflammation in susceptible patients or with delirium per se. The ability to remain independent is vital to many elderly patients as highlighted in a study by Salkeld et al., where 80 % of elderly female patients stated that they would rather die than lose their independence and be admitted to a nursing home establishment [15]. Emerging evidence now suggest that some delirium can persist, leading to poor outcomes and increased risk of dementia and cognitive dysfunction [8, 10, 13, 16–18]. There is no known effective treatment for delirium, creating the urgent need for research into delirium prevention.

It is estimated that there are currently 70,000–75,000 hip fractures per year in the United Kingdom (UK), and this is projected to increase by 2 % every year [19]. This represents a major burden on the National Health Service (NHS) responsible for an estimated 1.5 million bed days and cost of UK£2 billion per year. Elderly patients with hip fractures can be frail and have multiple health problems. National data revealed that the median length of stay in hospital is 23 days with 20 % of patients suffering complications post-operatively. Up to 8.3 % of patients die within 30 days of their surgery and approximately 30 % die 1 year after their surgery in England, with audit data showing a 7 % 30-day mortality and 18 % 120-day mortality in Scotland [20, 21]. These reports display small trends in reduction of early mortality, but there remains variability in patient outcomes in the UK. Hip fracture is also a worldwide problem with experts predicting the number of hip fractures occurring in the world each year rising to 6.26 million by 2050 [22].

Ninety-eight percent of patients with hip fractures are offered surgery and require anaesthesia [23]. Anaesthesia may be classified into general anaesthesia (GA) or regional anaesthesia (RA). In GA, patients are given either inhalational agents or intravenous anaesthetic agents to render them unconscious. RA is performed by numbing the nerves that supply sensation to the lower limbs, with the injection of local anaesthetic solution into the fluid surrounding the spinal cord (spinal) or by injection into the epidural space (epidural). When the operation site is numbed, the patient does not need to be unconscious during the operation.

One potential major benefit of RA is the avoidance of GA drugs and opiates which have been linked to post-operative delirium [24]. RA also allows earlier oral intake and quicker return to mobility such as those seen in enhanced recovery programme [25, 26]. Excessive depth of anaesthesia and perioperative hypotension under GA are associated with higher mortality [27]. Although RA removes the need for GA, perioperative hypotension can lead to cardiovascular instability [28, 29] and the use of sedation can also be overused in the elderly [30]. The Association of Anaesthetists of Great Britain and Ireland (AAGBI) and Scottish Intercollegiate Guidelines Network (SIGN) both cautiously recommend RA to be used for all patients unless contraindicated [23, 31].

There are a number of existing systematic reviews looking at anaesthesia in hip fracture patients. A Cochrane review by Parker et al. in 2004 discussed randomised controlled trials comparing regional and general anaesthesia in patients with hip fractures [32]. Twenty-two studies were included with seven reporting on ‘acute confusional state’. Data from five small studies concluded that regional anaesthesia might significantly reduce the risk of developing acute confusion by 50 % (*p* = 0.03). However, the studies that were included were small and excluded patients with cognitive dysfunction. The anaesthetic techniques compared in the studies were old and outdated. There was also a lack of detail on how acute confusion was detected and assessed in patients.

Other more recent systematic reviews did not focus on delirium as an outcome [33], did not consider the mode of anaesthesia [34] or only included randomised controlled trials (RCTs) [35, 36]. Common methodological limitations included restrictive selection criteria (exclusion of patients with cognitive impairment) and inadequate exploration of heterogeneity relating to rating scales used to diagnose/measure delirium and the time points at which this is recorded. The 2010 systematic review by Luger et al. assessed the effect of neuroaxial and general anaesthesia on the outcomes of morbidity and mortality in geriatric patients with hip fractures [28]. Their review included RCTs, observational studies and reviews/meta-analyses. However, their electronic literature searches were restricted to only PubMed and the Cochrane Library databases, from 1967 to 2010. Like the review by Abou-Setta et al., delirium/confusional state was not the primary outcome of interest [33].

Since the searches were undertaken for the systematic review by Luger et al. in 2010, a number of additional relevant studies have been published, notably the 2014 Anaesthetic Sprint Audit (ASAP) in hip fracture comparing the modes of anaesthesia [20, 37].

There is therefore a need for an up-to-date, methodologically robust systematic review to evaluate both experimental and observational evidence on the use of regional compared to general anaesthesia in patients undergoing hip fracture surgery, with a focus on delirium as an outcome. Many of the previous systematic reviews in this area included outdated anaesthetic techniques that are no longer relevant to clinical practice. The proposed review will therefore focus on those techniques/anaesthetics in current use to ensure relevancy of findings to this population. The proposed review will also examine the quality of delirium diagnosis and assessments, an aspect which has not been addressed in existing reviews but which is essential for assessing the validity of findings.

## Methodology

Reporting of this protocol followed the Preferred Reporting Items for Systematic Reviews and Meta-Analyses (PRISMA)-P guidelines [38]. The proposed review will use standard systematic review methodology aimed at minimising bias, and reporting will be in line with the PRISMA guidelines (http://www.bmj.com/content/349/bmj.g7647). This protocol is registered with PROSPERO (CRD42015020166).

A PRISMA-P file is attached (Additional file 1).

### Search strategy

A thorough and sensitive search strategy will be developed. Embase, MEDLINE, CINAHL and the Cochrane Library (CENTRAL) bibliographic databases will be searched from database inception to the present day. Our search term combinations will consist of index terms, text words and word variations for the concepts of population (hip fracture surgery) and intervention/comparator (all types of anaesthesia). The search strategy will be informed by a relevant Cochrane review by Parker et al. [32]. Clinical trial registers (www.clinicaltrials.gov and the International Clinical Trials Research Platform) will also be searched to identify any ongoing trials. The ZETOC and Web of Science websites will be searched to identify relevant conference proceedings. Authors of these trials will be contacted via e-mail and invited to contribute unpublished data. To complement the database searches, the bibliographies of all relevant primary articles and reviews will be searched by hand to identify any articles missed by the electronic searches. A comprehensive database will be constructed using EndNote 7.0 to store all identified references. No language restrictions will be applied. A sample search strategy can be found in (Additional file 2: Table S1).

### Study selection process

Studies will be selected in a two-step process. First, citations identified by electronic searches will be screened by title and, where available, abstract. Full-text manuscripts will be retrieved of those citations meeting or thought-to-be-meeting the pre-determined inclusion criteria. Two reviewers (JY, RC) will then independently inspect all the manuscripts to determine if they meet the following criteria:

### Inclusion criteria

- Population: any patient aged ≥60 (or where the majority are ≥60) undergoing surgery for fragility hip fracture.
- Intervention and comparator: any type of regional versus any type of general anaesthesia provided they are in current use such as those described in the UK AAGBI guidelines [23]. Studies comparing more than one type of regional and/or general anaesthesia will be included provided at least one type of each is being compared.
- Outcomes
  - Primary outcome
    - Post-operative delirium (POD) reported using any validated criteria, e.g. diagnosed using the DSM criteria or clinical reporting tools such as confusion assessment method (CAM), Delirium Rating Scale revised-98 (DRS-R-98) or Neelon and Champagne (NEECHAM) confusion scale [39].
  - Secondary outcomes
    - Mortality
    - Measures of functional outcome (including mobility, e.g. Barthel index, cumulated ambulation score)
    - Quality of life (e.g. SF-36, EuroQol Questionnaire (five domains) (EQ-5D))
    - Length of hospital stay
    - Discharge location
    - Adverse events as reported by the post-operative morbidity score (POMS) [40].
  - Studies will be included provided at least one of the outcomes is reported.
- Study design: randomised or non-randomised controlled trials, controlled studies (prospective or retrospective).

### Exclusion criteria

- Studies using anaesthetic agents or techniques considered not to be of standard practice, such as halothane, enflurane and xenon.
- Studies with patients undergoing hip fracture surgery alongside other orthopaedic surgeries, e.g. lower limb fracture.
- Uncontrolled studies.

### Data extraction

Data will be extracted on but not be limited to the following:

- Study characteristics (e.g. randomised or non-randomised, number of patients assigned to each group, number of patients lost to follow-up).
- Population characteristics (e.g. age, type of fracture, pre-existing cognitive impairment, use of morphine or additional regional block pre- and post-surgery).
- Intervention and comparator characteristics (e.g. type of anaesthetic used, timing of surgery, additional use of sedation).
- Outcomes (primary and secondary outcomes reported including effect sizes and uncertainty, direction of effect, diagnostic criteria/assessment tools used for assessing delirium, length of follow-up, discharge location, functional outcomes and quality of life).

Data will be extracted onto a pre-designed and piloted proforma. Any disagreements surrounding the selection of a manuscript or data extraction will be resolved either by consensus or arbitration by a third reviewer (JD or VP).

### Assessment of risk of bias and methodological quality

Quality assessment of the included studies will be tailored to the different study designs.

The ‘Cochrane Collaborations tool for assessing risk of bias’ will be applied to randomised controlled trials [41]. Each potential source of bias will be graded high, low or unclear with description of the decision documented. Where appropriate, this will be undertaken on an outcome-by-outcome basis. The following domains will be considered:

- Selection bias (sequence generation and allocation concealment).
- Performance bias (blinding of participants and personnel).
- Detection bias (blinding of outcome assessment).
- Attrition bias (incomplete outcome data).
- Reporting bias (selective reporting).

For prospective non-randomised controlled studies, quality assessment will also be based on the Cochrane risk of bias tool, with selection bias and potential baseline imbalances more likely to pose as risk of bias. These criteria will be supplemented where appropriate with those described in the Newcastle-Ottawa scale for (prospective and retrospective) cohort studies [42]. Potential confounding factors to consider include the use of sedation, the use of morphine and timing of surgery. Any information pertaining to the validity of assessment tools used will be extracted. Risk of bias across all studies will be narratively described and tabulated. The possibility of formal sensitivity analysis according to the study quality will be explored. Quality assessment will be undertaken in duplicates, alongside data extraction.

### Data synthesis

All findings will be described narratively and tabulated. Presentation of findings will be structured according to outcome and study design.

Clinical and methodological heterogeneity will be assessed based on study design, population, intervention and comparator characteristics and outcomes. This data will inform whether pooling of results in one or more meta-analyses is feasible. Heterogeneity may arise from differences in age, timing of surgery and use of sedation, and it is anticipated that subgroup analyses will be performed according to these variables (60–80 years of age vs. older/>80 years; timing of surgery early/<36 h since admission vs. late/>36 h since admission and use of sedation (regional anaesthesia vs. regional anaesthesia + sedation)). Additionally, if delirium risk stratification tool is used, then subgroup analysis will be conducted to compare high-risk patients with those deemed at low risk of developing delirium. Statistical heterogeneity will be assessed using the chi-square test and quantified with the *I*^2^ statistic. Data from RCTs and non-RCTs will be meta-analysed separately.

Delirium will likely be assessed using a variety of continuous measures (scales), e.g. for severity of delirium or there may be a definitive diagnosis of delirium (e.g. using the DSM criteria). Where a variety of continuous measures are used, the use of the standardised mean difference (SMD) in meta-analysis will be considered; alternatively, where there are a number of studies using the same measurement scale, these may be grouped together in separate meta-analyses. Mortality data will be presented as (pooled) relative risk (RR) where possible. Where possible, meta-analysis will be conducted using Review Manager (RevMan version 5.3) software. Methods of delirium assessment in the included studies will be described if reported. A detailed analysis of validity of each tool is beyond the scope of this review. Heterogeneity in delirium assessment will be taken into account during data reporting and meta-analysis.

In assessing the overall quality and strength of the evidence, the GRADE framework will be considered [43]. This will include an assessment of publication bias where feasible, e.g. using a funnel plot where there are ≥10 studies in a meta-analysis.

## Discussion

With an ageing population, hip fractures are likely to have a significant burden on public health [44]. It is increasingly recognised that post-operative delirium has a significant impact on patient outcome. Delirium is associated with increased mortality, morbidity, institutionalisation and poor functional outcomes [45, 46]. The pathophysiology of delirium remains poorly understood, but studies have highlighted some pre-existing and precipitating factors that may pre-dispose to post-operative delirium [5, 47, 48]. Previous systematic reviews have examined the choice of anaesthetic techniques (general anaesthesia or regional anaesthesia) as a probable modifiable factor that can impact on the development of post-operative delirium.

This systematic review will provide an updated evidence base using data from both RCTs and non-RCTs, with which to guide clinical practice and research for this group of challenging patients.

Previous systematic reviews are either out of date, excluded non-RCT evidence or did not consider delirium as a primary outcome measure of interest. Further limitations also relate to the exclusion of those with existing cognitive impairment or confusion who represented a large proportion of patients and as a result, severely reduced the generalizability of their findings. This updated review will focus on delirium and, in contrast to previous reviews, will also include a review of the assessment tools used to measure delirium. In order to maximise relevance, it will also exclude anaesthetic techniques and practices that are outdated and no longer in use in the UK.

Depending on whether we are able to demonstrate that the type of anaesthesia, i.e. ‘general’ or ‘regional’ anaesthesia, impacts on delirium and subsequent long-term outcomes, our review findings may support the need for a well-designed randomised control trial to assess the type of anaesthesia for hip fracture surgery on post-operative delirium and long-term outcomes.

## Additional files

Additional file 1: PRISMA Checklist. (DOCX 92 kb) Additional file 2: Table S1. Sample search strategy. (DOCX 63 kb)

## Abbreviations

AAGBI: Association of Anaesthetists Great Britain and Ireland
ASAP: Anaesthesia Sprint Audit of Practice
CAM: confusion assessment method
DRS-R-98: Delirium Rating Scale revised-98
DSM: Diagnostic and Statistical Manual of Mental Disorders
EQ-5D: EuroQol Questionnaire (five domains)
GA: general anaesthesia
NEECHAM: Neelon and Champagne
NHS: National Health Service
NIHR: National Institute of Health Research
POD: post-operative delirium
POMS: post-operative morbidity score
PRISMA: Preferred Reporting Items for Systematic Reviews and Meta-Analyses
RA: regional anaesthesia
RCT: randomised controlled trial
SIGN: Scottish Intercollegiate Guidelines Network
SMD: standardised mean difference
UK: United Kingdom
