# Supplementary material for: Regional versus general anaesthesia in elderly patients undergoing surgery for hip fracture: protocol for a systematic review
Source: Syst Rev. 2016 Apr 21;5:66. doi: 10.1186/s13643-016-0246-0 (PMC4839155; doi:10.1186/s13643-016-0246-0)
Supplement: Additional file 2: Table S1. — Sample search strategy. (DOCX 63 kb) [file 13643_2016_246_MOESM2_ESM.docx]

Table 1 Sample Search Strategy

Database: Ovid MEDLINE(R) <1946 to May Week 3 2015>

Search Strategy:

--------------------------------------------------------------------------------

1     exp Hip fracture/

2     hip fracture.mp.

3     (fracture$ adj2 (hip or femur$ or femor$)).tw.

4     or/1-3

5     exp an$esthesia/

6     an$esthesia.mp.

7     (anesthe$ or anaesthe$).tw.

8     an$ethetic.mp.

9     exp anesthetics/

10     exp general an$esthesia/

11     general an$esthesia.mp.

12     Anesthesia/

13     exp Anesthesia, General/

14     general an$esthesia.mp.

15     sedation.mp.

16     exp regional an$esthesia/

17     regional an$esthesia.mp.

18     peripheral an$esthesia.mp.

19     central blockade.mp.

20     central block.mp.

21     exp spinal an$esthesia/

22     spinal an$esthesia.mp.

23     exp epidural an$esthesia/

24     epidural an$esthesia.mp.

25     exp local an$esthesia/

26     local an$esthesia.mp.

27     infiltrative an$esthesia.mp.

28     peripheral nerve block.mp.

29     intravenous regional an$esthesia.mp.

30     systemic local an$esthesia.mp.

31     exp nerve block$/

32     nerve block$.mp.

33     neuroaxial blockade.mp.

34     Anesthesia/ or exp Anesthesia, Intravenous/

35     exp inhalation an$esthesia/

36     inhalation an$esthesia.mp.

37     or/5-36

38     4 and 37
